# Prelacteal feeding and associated factors among mothers having children less than 24 months of age, in Mettu district, Southwest Ethiopia: a community based cross-sectional study

**DOI:** 10.1186/s13104-019-4044-3

**Published:** 2019-01-07

**Authors:** Tarekegn Fekede Wolde, Amare Demsie Ayele, Wubet Worku Takele

**Affiliations:** 1 Department of Nursing, Faculty of Public Health and Medical Sciences, Mettu University, Metu, Oromia Region Ethiopia; 2 Department of Pediatrics and Child Health Nursing, School of Nursing, College of Medicine and Health Sciences, University of Gondar, Gondar, Ethiopia; 3 Department of Community Health Nursing, School of Nursing, College of Medicine and Health Sciences, University of Gondar, Gondar, Ethiopia

**Keywords:** Ethiopia, Mother–child pairs, Prelacteal feeding

## Abstract

**Objective:**

Despite prelacteal feeding contravenes with exclusive breastfeeding, it is a prevailing problem in Ethiopia. However, its burden and factors were not investigated in Mettu district. Therefore, the objective of our study was to conduct the burden of prelacteal feeding and its associated factors. Community-based cross-sectional study was conducted among 730 mother–child pairs. Stratified cluster sampling was used. Data were collected by face- to- face interview. Logistic regression model was fitted.

**Results:**

A total of 719 mother–child pairs with a response rate of 98.5% were participated. The overall proportion of prelacteal feeding among mothers was 14.2% [95% CI (12.0, 17.0)]. No maternal education [AOR: 3.54 (95% CI 1.7, 6.98)], single ANC visits [AOR: 6.87 (95% CI 3.21, 14.73)], didn’t know risks of prelacteal feeding [AOR: 2.73 (95% CI 1.47, 5.05)], colostrums avoidance [AOR: 6.030 (95% CI 3.48, 10.46)], home delivery [AOR: 3.04 (95% Cl 1.60, 5.75] and cesarean delivery [AOR: 4.27 (95% CI 2.28, 7.99)] were significantly associated factors. Prelacteal feeding among mother–child pairs was high. Hence, increasing maternal education and institutional delivery are vital for prompt infant feeding.

## Introduction

Breast milk is the ideal meal for growth and brain development, and lifesaving nutrient for infants **<** 6 months of age [1, 2]. In contrary, worldwide, nearly 2 from 5 breastfed newborns receive fluid/foods in their earliest days of life [1, 3].

Irrespective of the reasons, provision of any fluid and/or semisolids to newborns before initiation of breast milk is considered as prelacteal feeding [3, 4]. It is the main cause of suboptimal breastfeeding which increases the risk of neonatal infections, deaths, diarrheal diseases and acute respiratory illnesses [1]. Similarly, it is associated with stunting [5] and early cessation of breastfeeding [6].

Despite this fact, in many regions of the globe including Ethiopia, significant proportion of mothers offer prelacteals to their newborns [7–15] in that the record is highest in Southeast and Central Asia [15–19], modest in Latin America accounting 22.9–40% [20], average in Sub-Saharan Africa that showed 32.2% [7], and Ethiopia’s ranges from 6.7 to 56% [3, 4, 10, 21–24]. In literatures, lack of maternal education [21, 25, 26], antenatal care (ANC) utilization [7, 21], home delivery [10, 23, 27], cesarean delivery [9, 20, 21], unknowing to risks of prelacteal feeding [3, 10], late initiation of breastfeeding [27] and colostrums avoidance [28] were found to be factors affecting prelacteal feeding.

In Ethiopian community, raw butter, plain water, cow milk and glucose solution are the most common prelacteals [3, 10, 21, 28]; and tradition or culture, perception of breast milk insufficiency and cleaning the gut are reasons of prelacteal feeding [7, 12].

Since 2004, Ethiopia has been implementing the Infant and Young Child Feeding (IYCF) strategy as a key component of child survival approach [29]. However, breastfeeding was suboptimal [4], and prelacteal feeding has been continued as deep-rooted problem [4, 10, 11, 22]. In the past 20 years, though there is reduction of child mortality in Ethiopia, half of the prevailing mortality happens during neonatal period [4]. Consequently, investigating the burden and determinants of this problem is paramount to promote implementation of IYCF thereby reducing neonatal mortality. Nonetheless, the study area has limited evidence on this regard. The study could serve as baseline information for researchers and input for policy makers.

## Main text

### Methods and materials

#### Study design, setting and period

A community based cross sectional study was conducted in Oromia Regional state, Illu Aba Bor Zone of Mettu district from April 10-March 10, 2018 G.C. Mettu District is 600 km southwest of Addis Ababa, the capital of Ethiopia.

#### Sample size and sampling procedure

A total of 730 mother–child pairs were recruited by single population proportion formula using Z-score at 95% confidence level (1.96); national proportion of prelacteal feeding (P = 28.92% = 0.2892), d = 0.04 (4% margin of error), D = design effect (1.5) and of 5% non-response rate.

Stratified cluster sampling technique was employed in that the study population was first stratified by residence as rural and urban kebeles. The residential stratification yields 29 rural and 3 urban kebeles. Each stratum (rural and urban) was taken as clusters. One urban and 7 rural kebeles were randomly selected using lottery method. All mother–child pairs living in selected kebeles were recruited thereby 595 taken from rural and 124 from urban. Target households with mother–child pairs were identified by family folder.

#### Data collection tool and procedure

Data were collected by face-to-face interview using pre-tested structured questionnaire. Five data collectors and 2 supervisors were recruited for data collection process. One day intensive training and attentive follow up were done.

##### Operational definition

*Prelacteal feeding (YES/NO)* Giving fluid and/or semisolid food to newborn except medicines, vitamins and minerals before initiation of breastfeeding [3, 4].

#### Data analysis

Data were entered using Epi-info version 7.1 and analyzed by SPSS version 22. Frequencies and proportions were used to summarize the variables. Logistic regression model was fitted. For each variable, odds ratio with 95% confidence interval were estimated. Variables with *p* value < 0.05 were considered statistically significant with prelacteal feeding.

### Results

#### Socio-demography

A total of 719 mother–child pairs with a response rate of 98.5% were included in the study. Of these, 82.80% were from rural, 361 (58.5%) were within 25–34 years having mean age of 28.79 (± 5.47) years. The mean age of index children was 10.35 (± 3.48) months with 43.40% ranged from 6 to 11 months. Nearly three-fourth 441 (72.8%) of participants were Oromo by ethnicity, 269 (37.4%) housewives by occupation, and 386 (53.7%) were Orthodox Christian by religion (Table 1).

**Table 1:** Socio-demographic characteristics of mothers with children less than 24 months of age in Mettu district, Oromia regional state, southwestern Ethiopia, 2018 (n = 719)

Variables	Frequency (No)	Percent (%)
Maternal age		
≤ 24	194	27.00
25–34	410	57.00
≥ 35	115	16.00
Ethnicity		
Oromo	514	71.50
Amhara	140	19.50
Gurage	51	7.10
Kefa	14	1.90
Maternal religion		
Orthodox Christian	386	53.70
Protestant	203	28.20
Muslim	127	17.70
Catholic	03	0.40
Family type		
Nuclear	571	79.40
Single parents	97	13.50
Extended	51	7.10
Maternal monthly income		
≤ 500	223	31.00
501–1000	149	20.70
1001–2000	145	20.20
> 2000	202	28.10
Maternal occupation		
Housewife	269	37.40
Students	34	4.70
Government employment	161	22.40
Merchants	207	28.80
Farmers	48	7.60
Age of index child		
0–5 months	247	34.30
6–11 months	312	43.40
12–23 months	160	22.30 100.00
Sex of index child		
Male	431	59.90
Female	288	40.10
Birth order of index child		
1st	161	22.40
2nd	247	34.40
3rd	173	24.10
≥ 4th	152	19.19
Perceived wt. of index child		
Small	171	23.80
Average	519	72.20
Large	29	4.00
Residence		
Rural	595	82.80
Urban	124	17.20
Educational status		
No education	71	9.90
Read and write	151	21.00
Primary education	206	28.70
Secondary education	225	31.30
College and above	66	9.20
Total		100.00

In our manuscript, Table 1 shows the sociodemographic characteristics of mother–child pairs in the study area. We have depicted high frequencies in text and the details of demographic data were presented in the table

#### Maternal healthcare characteristics

From all participants, 694 (96.1%) have utilized ANC services, of which 83 (11.95%) of women utilize ANC once only. About 74.80% of women were multiparous, 152 (21%) did not get breastfeeding counseling and 82.30% gave birth at health facilities. About 13.50% delivered by cesarean section and 641 (89%) of mothers assisted by health professionals (Table 2).

**Table 2:** Healthcare characteristics of Mothers having Children less than 24 months at Mettu District, Oromia regional state, southwestern Ethiopia, 2018 (n = 719)

Variables	Frequency	Percent (%)
Antenatal care (n = 719)^a^		
YES	694	96.20
NO	25	3.80
Number of ANC (n = 694)		
1 times	83	11.95
2–3 times	342	49.27
≥ 4 times	269	38.78
Parity (n = 719)		
1st	181	25.20
2–3	445	61.90
≥ 4	93	12.90
Breastfeeding counseling at ANC clinic (n = 694)		
YES	542	78.10
NO	152	21.90
Information from counseling (n = 563)^b^		
Benefit of breastfeeding	199	35.34
Positioning and attachment	54	9.60
Exclusive breastfeeding	254	45.12
Non-breastfeeding problem	44	7.80
Expressed breastfeeding	12	2.13
Place of delivery (n = 719)		
Health facility	628	82.30
Home	91	12.70
Mode of delivery (n = 719)^c^		
Cesarean Section	97	13.50
Vaginal delivery	622	86.50
Assistant during delivery (n = 719)		
Health professionals	641	89.20
TBA	78	10.80
		100.00

^a^ At least one visit; TBA: traditional birth attendant

^b^ Multipleanswers

^c^ Assisted/instrumental delivery is coded as vaginal delivery

#### Prelacteal feeding practice

Out of 719 mother-infant pairs, 102 (14.2%) [95% CI 12.0, 17.0] reported that they gave prelacteals to newborns before initiation of breastfeeding. Of these, about 39.22% gave fresh butter, 36.27% provided pure/plain water, 18.60% fed animal milk, and 5.90% used sugar solution. About 82.50% of mothers fed colostrums to their index child, whereas 17.5% of them avoided it. Nearly 38.4% said that colostrums cause abdominal cramp and diarrhea, 32% complained breast milk insufficiency, 28% fear maternal illness, and 1.6% reported child growth retardation as reasons of colostrums avoidance. Around 72.7% of newborns were initiated breastfeeding on time (≤ 1 h). Pertaining to knowledge of mothers on risk of prelacteal feeding: more than half (64.12%) mothers didn’t know the risks, 13.76% of mothers reported diarrhea, 11.83% of mothers claimed it slows child growth, 6.39% of them reported it causes infections, and 3.89% claimed child vomiting.

#### Factors associated with prelacteal feeding among mother–child pairs

The multivariable analysis showed that educational status, number of antenatal care visits, knowledge on risks of prelacteal feeding, colostrums avoidance, place of delivery, and mode of delivery were found to be statistically significant positively associated factors with prelacteal feeding among mother–child pairs. Sequentially interpreted as follows:

The odds of practicing prelacteals among mothers without formal education was 3.537 times higher as compared to those having formal education [AOR: 3.537 (95% CI 1.793, 6.978)].

The odds of practicing prelacteals among mothers attending single ANC visit was 6.872 times as compared to those attending four or more visits [AOR: 6.872 (95% CI 3.206, 14.729)].

The odds of practicing prelacteal feeding among mothers who are unknowing about risks of prelacteals was 2.725 times higher as compared to those knowing the risks [AOR: 2.725 (95% CI 1.472, 5.046)].

The odds of practicing prelacteals among mothers who avoid colostrums was 6.030times higher as compared to those who fed it [AOR: 6.030 (95% CI 3.478, 10.455)].

The odds of practicing prelacteal feeding among mothers experienced home delivery was 3.035 times higher as compared to those experienced institutional delivery[AOR: 3.035 (95% CI 1.603, 5.747)].

The odds of practicing prelacteal feeding among mothers experienced cesarean section was 4.266 times as compared to those experienced spontaneous vaginal delivery [AOR: 4.266 (95% CI 2.276, 7.996)] (Table 3).

**Table 3:** Bivariable and multivariable logistic regression analysis of factors associated with prelacteal feeding among mothers having children less than 24 months of age in Mettu district Southwestern, Ethiopia, 2018 (n = 719)

Variables/category/	Prelacteal feeding Yes No		COR (95% CI)	AOR (95% CI)
Educational status				
No formal	24 (33.80%)	47 (66.20%)	3.732 (2.162, 6.440)*	3.537 (1.793, 6.978)**
Formal	78 (12.04%)	570 (87.96%)	1.00	
Family type				
Nuclear	74 (12.96%	497 (87.04%)	1.00	1.975 (0.860, 4.537)
Single parents	14 (14.43%)	83 (85.57%)	1.33 (0.611,2.099)	.837 (0.376, 1.861)
Extended	14 (27.45%)	37 (72.55%)	1.487 (1.087, 2.033)*	
Sex of index child				
Male	72 (16.71%)	359 (83.29%)	0.580 (.368, .914)*	1.500 (0.864, 2.605)
Female	30 (10.42%)	258 (89.58%)	1.0	
Residence				
Rural	92 (15.46%)	503 (84.54%)	0.480 (0.242, 0.950)*	1.908 (0.827, 4.404)
Urban	10 (8.06%)	114 (91.94%)	1.00	
Number of ANC				
1st	35 (42.17%)	48 (57.83%)	0.468 (0.365, 0.600)*	6.872 (3.206, 14.72)**
2–3	44 (10.02%)	294 (86.98%)	2.192 (1.222,3.933)*	
≥ 4	17 (6.39%)	249 93.61%)	1.00	
Counseling on breastfeeding				
Yes	59 (10.41%)	508 (89.59%)	1.00	1.529 (0.809, 2.889)
No	43 (28.29%)	109 (71.71%)	3.397 (2.179, 5.296)*	
Know risks of prelacteal feeds				
Yes	16 (6.20%)	242 (93.79%)	1.00	2.725 (1.472, 5.046)**
No	86 (18.65%)	375 (81.34%)	3.469 (1.986, 6.058)*	
Colostrums avoidance				
Yes	52 (41.27%)	74 (58.73%)	7.631 (4.828, 12.06)*	6.030 (3.478, 10.45)**
No	50 (8.43%)	543 (91.57%)	1.00	
Assistant during birth				
Health personnel	78 (12.17%)	563 (87.83%)	1.00	0.318 (0.066, 1.529)
TBA	24 (30.77%)	54 (69.23%)	3.208 (1.877, 5.483)*	
Place of delivery				
Health facility	71 (11.31%)	557 (88.69%)	1.00	3.035 (1.603, 5.747)**
Home	31 (34.06%)	60 (65.93%)	4.053 (2.461, 6.676)*	
Mode of delivery				
Cesarean section	28 (28.86%)	69 (71.13%)	3.005 (1.819, 4.963)*	4.266 (2.276, 7.996)**
Vaginal delivery	74 (11.89%)	548 (88.01%)	1.00	

*COR* crude odd ratio, *AOR* adjusted odd ratio, *CI* confidence interval

*Associated by binary logistic regression with p-value < 0.05, ** Associated by multivariable logistic regression with p-value < 0.001

### Discussion

In this study, the overall proportion of prelacteal feeding among mother–child pairs was found to be 14.2% (95% CI 12.0, 17.0). It is in line with studies of Jimma 12.6% [30], and Tigray 12.8% [31]. However, it is higher than studies elsewhere in Ethiopian: East Wollega 6.7% [32], North Wollo 11.1% [28] and Arba Minch 8.9% [33]. The discrepancy could from the current study gives due emphasis for both rural and urban residence whereas those studies focuses only rural residence. The finding was lower than studies of Raya kobo 38.8% [10], Harari 45% [11] and Dabat 56% [22]. This is stemmed from socio-cultural difference. It also lower than studies from Egypt 58% [12], Vietnam 73.3% [15] and India 49.5% [18] which emanated from the difference in maternal beliefs and attitude towards breastfeeding and socio-cultural variations.

The odds of providing prelacteals among uneducated mothers were nearly four folds higher than who were educated. This was supported by evidences [27, 34, 35] as in maternal education increases utilization of healthcare services and positively affects proper infant feeding practices.

Mothers having single ANC visit practiced prelacteals approximately seven times higher than who followed 4 or more. This was congruent with study reports [7, 26]. As the number of ANC visits increase, the likelihood of getting lactation counseling on infant and child feeding will be increased and promoted.

The odds of practicing prelacteal feeding among cesarean delivered mothers were nearly four times higher compared to vaginally delivered mothers. Evidences like [7, 9, 18, 19, 21] showed that C/S influences the family, tradition/custom/of the community and fear of pain after delivery that delays breastfeeding initiation which in turn facilitates use of prelacteals. A meta-analysis showed that cesarean section delays initiation of breastfeeding [16].

Home delivery increases prelacteal feeding by three folds than institutional delivery. This was congruent with studies [10, 28, 34]. In this study, nearly half (51%) of mothers were influenced by colleagues, traditional birth attendants and husband while delivering at home that facilitate prelacteal feeding; whereas encouraged to avoid prelacteals during postnatal care services at healthcare.

Colostrums avoiding mothers had six times odds of practicing prelacteal feeding compared to colostrums feeding counterparts. This was parallel with studies [23, 28] as in there may be influence from the traditional birth attendants. Moreover, maternal misperception and/or misconception that colostrums may cause newborn sickness and considering it as dirty milk are the reasons of avoiding this medicinal substance [6, 15].

The odds of practicing prelacteals among mothers who didn’t know the risks of prelacteal feeding were nearly 3 times higher than their counterparts. This was similar with studies [3, 10, 15] that could be explained as none and/or low maternal education and reduced ANC visits have negative impacts on proper infant feeding.

### Conclusion

Prelacteal feeding was common in the study area; and found to be high. Thus, emphasis should be given to improve awareness on risks of prelacteal feeding, benefits of colostrums feeding, ANC follow up visits and increases institutional delivery services.

## Limitations

Though standard data quality assurance mechanisms were applied, there might be a chance of recall and information biases in ascertaining some of the variables, such as healthcare access and time to initiation of breast milk.

## Abbreviations

ANC: antenatal care
PLF: prelacteal feeding
SPSS: statistical package for social sciences
WHO: World Health Organization
IYCF: Infant and Young Child Feeding
SD: Standard deviation
